# Chemoprophylaxis and Malaria Death Rates

**DOI:** 10.3201/eid1203.050736

**Published:** 2006-03

**Authors:** Gérard Krause, Irene Schöneberg, Doris Altmann, Klaus Stark

**Affiliations:** * Robert Koch-Institute, Berlin, Germany

**Keywords:** malaria, chemoprophylaxis, CFR, chloroquine, plasmodium falciparum, research

## Abstract

To determine the effect of chemoprophylaxis on the case-fatality rate of malaria, we analyzed all cases of *Plasmodium falciparum* malaria in nonimmune persons reported from 1993 to 2004 in Germany. In univariate and multivariate logistic regression analysis, we determined the effect of age, sex, chemoprophylaxis, chemoprophylactic regimen, compliance for chemoprophylactic regimen, exposure prophylaxis, country of infection, and year of reporting on the outcome. Of 3,935 case-patients, 116 (3%) died of malaria. Univariate analysis showed significant associations with death for chemoprophylaxis with chloroquine plus proguanil compared to no chemoprophylaxis. The multivariate model showed that patients who had taken chemoprophylaxis were less likely to die compared to those who had not taken chemoprophylaxis, adjusted for patient age and reporting year. The study demonstrated that chemoprophylaxis significantly reduced fatality rates among nonimmune malaria patients and supports the importance of existing guidelines for malaria prevention.

## Abstract 2

Malaria chemoprophylaxis increases the survival of nonimmune travelers.

The estimated risk of nonimmune travelers to malaria-endemic countries acquiring malaria is 1–357 per 100,000 depending on endemicity of the country (*1*). Approximately 800 imported malaria cases are reported through the notifiable disease surveillance system in Germany each year, about twice as many per population as in the United States (*2*). Within the World Health Organization European Region, Germany is the country with the third largest number of reported imported malaria cases following France and the United Kingdom (*3*).

Exposure prophylaxis (repellents and bed nets) and chemoprophylaxis are established methods of preventing malaria during travel in malaria-endemic countries; its importance has recently been underlined by Chen and Keystone as well as by Zuckerman (*4**,**5*). Persons from nonendemic countries are considered nonimmune because their risk of acquiring malaria and subsequently developing severe disease with possible fatal outcome is considerably higher than for adults who have spent their childhood in a malaria-endemic environment (*6*).

The lack of randomized controlled trials on the effectiveness of chemoprophylaxis on appropriately characterized travelers has been rightly criticized, yet the methodologic difficulties of realizing such investigations are obvious (*7*). Alternative study designs based on surveillance data may provide some evidence whether nonimmune travelers to malaria-endemic countries would have benefited from chemoprophylaxis even if it had failed to prevent the disease. The strongest outcome measure for this question is the case-fatality ratio (CFR). Because fatal malaria is rare in nonendemic countries, various studies on imported malaria have not had the statistical power to investigate the case-fatality rate under inclusion of relevant confounders (*8**,**9*). Multinational networks able to overcome the problem of small sample size collect their data from specialized centers, causing a number of selection biases that may have particular impact on the CFR (*8**,**10*).

## Methods

From 1993 to 2000, physicians and laboratories in Germany reported malaria cases to local health departments, which then sent special case report forms to the Robert Koch Institute, the federal agency for infectious disease control. The forms contain information on age, sex, travel history, *Plasmodium* species, prophylactic measures, onset of disease, and death. Since 2001, after new legal requirements, laboratories and physicians report directly to the Robert Koch Institute. The report forms have had only minor changes over the years, which ensures comparability of the data.

The study was limited to reported *Plasmodium falciparum* malaria in persons from nonendemic countries. A case of *P. falciparum* malaria was determined when *P. falciparum* was directly detected in a person's blood. All cases of mixed infections containing *P. falciparum* and another subspecies were removed from the analysis.

Endemicity of a country was determined by using the World Health Organization's list of malaria-endemic countries (*11*). Only persons with German nationality or origin or cases originating from other nonendemic countries were considered nonimmune and included in the study. Country of travel was defined as the malaria-endemic country in which the patient stayed during the incubation period. If >1 country was named, the region or continent to which all countries belong was used.

Death was used as the outcome variable. The following confounding variables were considered for the analysis: age, sex, year of reporting, chemoprophylaxis, chemoprophylactic regimen, patient compliance for chemoprophylaxis, exposure prophylaxis (repellents and bed nets), and country of travel. All but the first 3 variables were assessed by patient history. Information on type of treatment and time between onset of symptoms and treatment was not included in the analysis as it was not consistently available throughout the study period.

For univariate and multivariate logistic regression analysis, we used SPSS version 13.0 (SPPS Inc., Chicago, IL, USA). The method for variable selection was forward stepwise (using likelihood ratio statistics) taking into account all variables listed in Table 1. The confidence interval (CI) for all analysis was 95%.

**Table 1:** Univariate analysis of risk factors for fatal outcome of imported *Plasmodium falciparum* malaria in nonimmune patients, Germany 1993–2004

Risk factor	Odds ratio	95% confidence interval	p value
Chemoprophylaxis, total (n = 3,752)			
No	1		
Yes	0.743	0.493-1.121	0.157
Chemoprophylaxis, comparison of regimens (n = 3,752)			0.047
None (n = 2,171)	1		
Chloroquine alone (n = 485)	1.372	0.824-2.285	0.225
Proguanil alone (n = 59)	0.550	0.075-4.030	0.556
Mefloquine alone (n = 322)	0.503	0.201-1.258	0.142
Chloroquine plus proguanil (n = 459)	0.280	0.102-0.773	0.014
Other (n = 256)*	0.765	0.328-1.784	0.536
Compliance of chemoprophylaxis (n = 3717)			0.293†
No chemoprophylaxis	1		
Chemoprophylaxis with incomplete or unknown compliance	0.829	0.535-1.284	0.401
Chemoprophylaxis complete	0.538	0.231-1.249	0.149
Age (n = 3,844)			
Continuously (by year)	1.055	1.040-1.070	<0.001
Sex (n = 3,901)			
Female	1		
Male	1.141	0.768-1.695	0.515
Country of infection in Africa (n = 3,832)			
No	1		
Yes	3.642	1.150-11.529	0.028
Reporting year (n = 3,935)			0.004†

*235 were combinations of drugs that are not officially recommended regimens, 15 were doxycycline alone, and 6 were atovaquone/proguanil. †Overall p value for the categoric variable.

## Results

From 1993 to 2004, the Robert Koch Institute received reports on 6,964 cases of *P. falciparum* malaria, 2,371 cases due to other species or mixed infections, and 521 cases due to unidentified species. Among the cases of *P. falciparum* malaria, 3,935 (57%) patients were nonimmune and included in the subsequent analysis. A total of 116 patients in this study population died, resulting in a CFR of 3% (Table 2). Chemoprophylaxis was taken by 1,581 (42%) of the 3,752 persons for whom this information was available. The proportion of person who took chemoprophylaxis declined over the years (Figure). Univariate analysis of risk factors is shown in Table 1. Variables not shown in these tables were not significantly associated with death in any of the analyses.

**Table 2:** Imported *Plasmodium falciparum* malaria among nonimmune persons in Germany, 1993–2004

Year	No. cases	No. deaths (%)
1993	258	5 (1.94)
1994	419	19 (4.53)
1995	349	15 (4.30)
1996	412	13 (3.16)
1997	406	9 (2.22)
1998	378	19 (5.03)
1999	428	20 (4.67)
2000	326	2 (0.61)
2001	312	7 (2.24)
2002	232	2 (0.86)
2003	227	3 (1.32)
2004	188	2 (1.06)
Total	3,935	116 (2.95)

**Figure:**
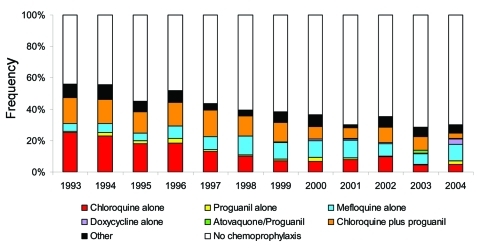
Proportional frequency of chemoprophylactic regimen taken by nonimmune patients of imported *Plasmodium falciparum* malaria, Germany 1993–2004.

Univariate analysis showed that increasing age and infection acquired in Africa were positively associated with fatal outcome. Chloroquine plus proguanil was inversely associated with fatal outcome compared to no chemoprophylaxis. The year of reporting was significantly associated with fatal outcome but did not show a linear association.

The results of multivariate analysis are shown in Table 3. In contrast to the univariate analysis, chemoprophylaxis was significantly associated with death. Age and reporting year remained significantly associated in the multivariate model (Table 3).

**Table 3:** Multivariate analysis of risk factors for fatal outcome of imported *Plasmodium falciparum* malaria in nonimmune patients, Germany 1993–2004

Risk factor (N = 3,681)	Odds ratio	95% confidence interval	p value
Chemoprophylaxis			
No	1		
Yes	0.629	0.403-0.983	0.042
Age	1.055	1.039-1.070	<0.001
Reporting year			0.003*

*Overall p value for the categoric variable.

## Discussion

This study demonstrated an independent effect of chemoprophylaxis on fatal outcome. For nonimmune patients with *P. falciparum* malaria who had taken any chemoprophylaxis (adjusted for age and reporting year), the risk of dying of the disease was two thirds that of those who had not taken any chemoprophylaxis (odds ratio [OR] 0.63, 95% CI 0.40–0.98). We are not aware of any such association being reported. Yet the findings are consistent with earlier reports (*9**,**12*). Our findings are also in line with observations made in numerous case reviews in which severity of illness appeared to be lower among patients who had taken chemoprophylaxis compared to patients who had not (*13**–**15*). Fatal outcome could be seen as the consequence of severe malaria, which in turn is associated with high parasitemia (*9**,**13*). If unable to prevent infection, chemoprophylaxis would likely slow down the parasite growth rate, which would result in a larger window of opportunity in which treatment might prevent death (*14**,**16*). Our data suggest that even in cases where chemoprophylaxis fails to prevent the development of malaria, it still significantly reduces the risk of dying from it. This finding may be important for travelers to malaria-endemic countries, adding another good reason to take chemoprophylaxis, in addition to reducing the risk of acquiring the disease.

Our study was also able to individually analyze specific chemoprophylactic regimens and identify significant associations for some of the individual regimens. In the univariate analysis, the risk of dying from malaria for patients who had taken the combination of chloroquine plus proguanil as a chemoprophylaxis regimen was less than one third that of those patients who had not taken any chemoprophylaxis (OR 0.28, 95% CI 0.10–0.77).

Chemoprophylaxis with doxycycline, atovaquone/proguanil, mefloquine, or proguanil did not show a significant association. This finding may be because the smaller prevalence of these regimens may have resulted in insufficient statistical power and does not necessarily question the prophylactic effectiveness of these regimens (*7*).

We can assume that recommendations for chemoprophylaxis were quite similar at any given point in time, since our study population was limited to Germany, and they agree with the current recommendations in the United States and the United Kingdom (*5**,**17**–**21*). The risk for infection, particularly the prevalence of chloroquine-resistant *P. falciparum*, has changed over the years in some endemic regions, and our study design has partly controlled for this by including the reporting year into the model.

The analysis also showed that increasing age was an independent risk factor for death. Age has been identified as a risk factor for severe disease or fatal outcome of malaria in several studies and case reports from the United States, Europe, and Israel (*8**,**9**,**12**,**15**,**16**,**22**,**23*). In contrast to those previous studies, we decided not to group the age into categories because our study population was sufficiently large to use age as a continuous variable, which allowed us to avoid any kind of classification bias. Our findings confirmed that the risk of dying from malaria increases with age (OR 1.06, 95% CI 1.04–1.07). As discussed by Mühlberger et al., the most likely explanation for the observed age effect is that with increasing age the immune system looses its capacity to generate a competent immune response against previously unencountered pathogens (*12*). Our study adds an important conclusion to this finding: although the elderly have an increased risk of dying from malaria, they can significantly reduce this risk by taking chemoprophylaxis.

The reporting year proved to be significantly associated with the CFR. We controlled for it by including it in the model as a categoric variable, since the association was not a linear one. We recommend that controlling for the year of data collection should also be considered in similar analyses of data collected over an extended period of time.

Although a technical change in the reporting mechanism occurred in 2001, it is not likely associated with the observed change of CFR; the decline in CFR was already observed before 2001, and the national death registry also showed a parallel decline of malaria deaths (*24*). From 1989 to 1995, CFR for all cases has generally been higher in Germany (3.6%) than in several other European countries and the United States (≈1%) (*25*). Meanwhile, CFR in Germany has declined to <1%. This decline may have been caused by a combination of better prophylactic regimens, improved pretravel counseling, chemoprophylaxis compliance, and earlier diagnosis and treatment. The treatment delay and type of treatment, in particular, might have played a role. Although this information was not consistently available in the study population, reporting forms have been changed so that future analyses should provide some evidence for or against this hypothesis. Additionally, physicians and pharmacies have begun providing pretravel advice, which may have affected the aforementioned factors. Providing this advice in the past has been the domain of a few highly specialized centers (*26*).

The study was focused on nonimmune patients, which were identified by their nationality or citizenship (until reporting year 2000) or by country of origin (from reporting year 2001 onwards). Both variables serve as a proxy for non-immunity and have been used as such in previous studies (*12**,**15*). During a transition period from 1999 to 2000, nationality and country of birth were simultaneously assessed in our surveillance system, and a comparison of both variables showed that the discrepancy was ≈5%. Therefore, we do not expect this technical change to have any relevant impact on our findings. Legal constraints do not allow collecting information on ethnicity or more detailed information on the geographic origin of a person in Germany.

In contrast to studies based on single institutions or networks of specialized centers, our study population is representative in that it included cases identified by any laboratory regardless of where and how the patient was treated. This strategy reduces the risk for selection bias, which is of particular importance when studying CFR.

The univariate analysis indicates that malaria acquired in Africa has a higher CFR than malaria acquired elsewhere (*10**,**13*). Lewis et al. have shown that severe malaria was observed more commonly in patients returning from countries in central, southern, and eastern Africa compared to those returning from countries in western Africa (*15*). However, risk assessment with reference to the country of infection is problematic, as reliable denominator data on exposure are difficult to obtain, often do not take the duration of exposure into account, and may not be reliable (*25**,**27**–**30*).

While chemoprophylaxis clearly reduces the risk of acquiring malaria in nonimmune persons, the travelers' compliance in taking chemoprophylaxis is quite variable (*3**,**30**–**32*). Depending on the country and the method of assessment, the proportion of malaria patients who take chemoprophylaxis is 19%–90% and has repeatedly been identified as a major limitation of preventing imported malaria (*2**,**4**,**14**,**33**,**34*). Like the recent publication by Askling et al. (*1*), this work demonstrates how data originating from notifiable disease surveillance may lead to research results with important clinical implications, therefore underlining the importance of such surveillance systems. We demonstrated that chemoprophylaxis significantly increases the chance of nonimmune patients to survive imported *P. falciparum* malaria. We suggest that this information be used in pretravel counseling to further motivate persons traveling in malaria-endemic countries to comply with recommended chemoprophylactic regimens.
